# The how and why of producing policy relevant research: perspectives of Australian childhood obesity prevention researchers and policy makers

**DOI:** 10.1186/s12961-021-00687-0

**Published:** 2021-03-10

**Authors:** Robyn Newson, Lucie Rychetnik, Lesley King, Andrew J. Milat, Adrian E. Bauman

**Affiliations:** 1 School of Public Health, University of Sydney, Sydney, Australia; 2 The Australian Prevention Partnership Centre, Sax Institute, Sydney, Australia

**Keywords:** Research impact, Policy, Research translation, Research funding, Public health, Childhood obesity

## Abstract

**Background:**

Understanding why research is conducted may help address the under-utilisation of research. This study examined the reasons for childhood obesity prevention knowledge production in New South Wales (NSW), Australia, and the factors influencing research choices from the perspective of the researchers and health policy agencies contributing to the research.

**Methods:**

A literature search of SCOPUS and ISI Web of Knowledge (affiliation and key word searches) was conducted to compile a database of NSW childhood obesity research outputs, published between 2000 and 2015 (*n* = 543). Descriptive statistics were used to quantify outputs by research type, differentiating measurement, descriptive, and intervention research, systematic reviews and other publications. Interviews were conducted with a sample of researchers drawn from the database (*n* = 13) and decision makers from health policy agencies who funded and contributed to childhood obesity research in NSW (*n* = 15). Researcher interviews examined views about societal impacts, why and under what circumstances the research was conducted. Decision-maker interviews examined policy agency research investment and how research was used in decision making. Content analysis and a thematic approach was used to analyse the interview transcripts.

**Results:**

The research in this case was conducted for mix of reasons including those traditionally associated with academic inquiry, as well as intentions to influence policy and practice. Differences in funding mechanisms, administrative and employment arrangements, and ‘who’ initiated the research, created differing incentives and perspectives for knowledge production. Factors associated with the characteristics and experience of the individuals involved also influenced goals, as did the type of research conducted. Policy agencies played a role in directing research to address policy needs.

**Conclusions:**

The findings of this study confirm that researchers are strongly influenced by their working environment. Funding schemes and other incentives to support policy relevant knowledge production are important. Contextual factors such as policy priorities, policy-driven research funding and the embedded nature or strong connections between some researchers and the policy agencies involved, are likely to have influenced the extent to which policy goals were reported in this study.

## Background

The gap between research and policy and the under-utilisation of research have been the subject of substantial commentary and research activity spanning over 50 years [1–5]. Studies consistently find that the quality of research, its relevance and accessibility are important factors in research use by policy makers [1, 2]. Yet, public health policy-makers commonly report the research available to them does not provide the information needed for decision-making [6]. This view is supported by studies examining publication outputs and content. Publication outputs focussing on describing or understanding public health problems or developing solutions are more common than those providing information about how to deliver or adapt these solutions under real-world circumstances [7, 8]. Systematic reviews rarely provide information required to make policy-decisions, such as information about the generalisability, adaptability or cost of interventions [9].

Considering ‘usability’ or the ‘societal value’ of research may increase its relevance to decision-makers [10, 11]. Studies examining researchers’ reasons for conducting their research report tensions between academic pressures and desire for research to have a positive real-world impact [12, 13]. These tensions are also reflected in academic hiring practices, which currently favour traditional evaluation criteria associated with research quantity and quality in comparison to criteria associated with external engagement and research translation [14, 15]. Existing studies suggest the relative emphasis researchers place on societal versus academic goals depends on a range of factors including the type of research being conducted (e.g. basic research versus applied research) [16–18]. Contextual factors such as academic performance measures, research funding mechanisms [12, 17–19] and socially-orientated organisational conditions [19] also play a role. Finally, the individual characteristics of researchers, such as their experiences working with non-academic stakeholders and their academic seniority, are important influences [12, 19, 20].

Involvement of decision makers in co-creation of research has been linked to improvements in the relevance and use of research [11, 21, 22]. Interactive modes of knowledge production may facilitate appreciation and utilisation of research by decision makers and enhance researchers’ understanding of policy processes [21, 22]. Such models have been adopted by funding bodies, who are increasingly requiring health research proposals to include partnerships with decision makers [11]. Existing studies provide case study examples of such collaborations [11, 23–25] and go some way towards defining effective approaches in terms of their structure and purpose [11, 19, 23]. For example, research-policy partnerships may differ in terms of the nature and stage of involvement of decision-makers in the research process, as well as the formality, length and complexity of relationships [11, 19]. Other studies focus on translation mechanisms across research systems [26–28]. Here a distinction is made between: (1) producer-push efforts, led by researchers and research funding bodies (e.g. as part of grant assessment criteria); (2) user-pull efforts by decision-makers (e.g. research agenda setting strategies and organisational support for research use, capacity building and participation in research); and (3) exchange efforts and linkage activities that bring research producers and users together (e.g. support for knowledge linkage organisations and knowledge brokers) [26, 27].

Such considerations are part of a broader shift in approaches to knowledge production, from a focus on curiosity-based scientific inquiry to increase knowledge, to approaches where greater emphasis is placed on the societal value of research [29]. This shift is described variously, but most commonly referred to as ‘mode 1′ (autonomous and public research institution-based science), and ‘mode 2′ (interdisciplinary and taking place in real-world rather than academic contexts) [19, 29]. Existing studies suggest mode 2 research is still emerging in health fields [26, 30–32], with its presence varying in degree or emphasis across research disciplines [26, 30–32]. Specific policy-driven mechanisms*, including incentives, priorities and other forms of ‘directing’ research* (p3) [19], may facilitate the movement from mode 1 to mode 2 approaches.

Studies describing knowledge production contexts occupy a relatively small niche within the research use literature, focussing on understanding why and how knowledge is produced rather than whether and how knowledge is translated and used. Therefore, theories and models describing research use that are commonly utilized in research impact and knowledge to action studies are not a good fit for studies investigating the circumstances of knowledge production [5, 31]). Instead, knowledge production studies draw on the literature explaining transformations in research approaches, for example, the shift from mode 1 to mode 2 approaches as described in the preceding paragraph [19, 23, 29]. They also use metaphors such as producer push and demand-pull to explain mechanisms at the research-policy interface that influence knowledge production [23, 27]. In addition, some studies draw on theories and models explaining the workings of the research-policy nexus [33]. These include actor-network theories (interactions between actors working in complex systems), theories about institutional re-design (changes in institutional rules influencing interactions and behaviours), and theories describing the blurring of boundaries between traditional academic and policy communities (adapted from earlier work differentiating these communities [4]) [33]. This is a new area of inquiry and no single approach to studying knowledge production contexts has been adopted.

In this paper, we examine the context of knowledge production in relation to childhood obesity prevention research conducted in New South Wales (NSW), Australia, between 2000 and 2015. At the start of our study period, childhood obesity prevention research was a developing research area; evidence was available concerning the magnitude of the problem, however, there was limited empirical evidence for many of the influencing factors and related interventions [34]. At the same time, childhood obesity prevention policy was in its infancy in Australia [35]. The NSW Department of Health developed a major policy response which commenced in 2002 with the convening of the NSW Childhood Obesity Summit [36, 37], and ‘Tackling Childhood Obesity’ remained a substantial policy priority at the conclusion of our study period [37]. Knowledge production and research infrastructure development were key aspects of the NSW Government policy response [38, 39]. There was also significant local research interest in this area during the study period [40]. This policy and research area was chosen for study as it provided an opportunity to examine the progression of local research, alongside the development of local public policy, both addressing a relatively new and pressing public health problem [41]. It also provided an opportunity to explore the relationship between research and policy communities working in close geographical proximity to each other, over an extended period of time.

In studying the childhood obesity prevention knowledge production context in NSW, our aim was to understand why the reported research was conducted, both from the perspective of researchers and health policy agencies who had contributed to the research. We were interested in how the context of knowledge production in NSW influenced research choices between primarily academic or policy and practice focussed research. This study contributes to the literature examining the links between research and policy, and to what extent researchers are aware of and influenced by the policy context. It also contributes empirically derived insights about the role of strategies designed to direct research and generate policy relevant knowledge, examining their influence on research agendas and activities.

## Methods

### Overview and research questions

A database of NSW childhood obesity prevention research outputs, published between 2000 and 2015, was developed for the study. The database quantified the type of research published during this period. It was used to identify the initial set of interview respondents (researchers) and guide interview discussions. Interviews were conducted with NSW researchers and population health policy decision makers to address the following research questions:

1. Why was the defined body of childhood obesity prevention research conducted in NSW between 2000 and 2015?
2. How did the knowledge production context in NSW influence childhood obesity prevention research choices?

### Research output database

The research output database was developed by searching SCOPUS and ISI Web of Knowledge. Affiliation field searches (university name) combined with key word searches (for ‘child’ and ‘obesity’ related terms; Box 1) were used to identify publications. Peer reviewed childhood obesity prevention research conducted in NSW or by NSW researchers between 2000 and 2015 was included. Publications were extracted, and their bibliographic details compiled and reviewed to determine if they met our inclusion criteria (Box 1). A website search of university, government and non-government sites was conducted to identify additional peer reviewed publications and ‘grey literature’ research reports for discussion in the interviews. Included peer-reviewed outputs were categorized using a typology differentiating measurement, descriptive, and intervention research, systematic reviews/meta-analyses and other types of publications as defined in Box 2 [7]. Descriptive statistics were utilized to describe the characteristics of published research.

Box 1: Search terms and inclusion criteria for peer reviewed research outputs Scopus and ISI Web of Knowledge database search: Affiliation field used to search each NSW research institution separately combined with key word search terms related to child and obesity (adolesc* or child* or juvenile or infant or youth AND obesity or overweight or ‘childhood obesity’)

Papers were included in the peer reviewed publication database if they met the following criteria:

1. Published between 2000 and 2015 in English
2. Published in a scientific journal (books and grey literature reports excluded)
3. Abstract or publication could be obtained for review
4. Title, abstract or keywords mention obesity or related terms (obesity, obese, overweight, BMI, body mass, weight, weight status, healthy weight, body weight, weight gain)
5. Study population included children and/or adolescents or publication referred to children and/or adolescents
6. At least one author affiliated with a NSW research institution (for non-data-based research) or research wholly or partially conducted in NSW (for data-based research)
7. Primary focus of the article was on obesity prevention, behaviour change interventions or prevalence/correlates of obesity (clinical e.g. pharmaceutical and surgical interventions, as well as, biomedical studies were excluded)

Supplementary website search of NSW tertiary research institutions, NSW Ministry of Health/Office of Preventive Health, local health districts, NSW Heart Foundation, and NSW Cancer Council, websites.

Box 2: Definitions of research type

**Table:** 

Descriptive research:	Describes the nature, scope and correlates of the problem
Intervention research:	Evaluates the impact of an intervention on health behaviours or outcomes, implemented under controlled conditions (efficacy studies), implementation under real world conditions in different samples (replication studies), or the wide scale use of an intervention (dissemination studies)
Measurement studies:	Development of measures or testing the reliability, acceptability or validity of measurement instruments
Research synthesis:	Meta-analyses; systematic reviews; literature reviews
Other:	Commentaries, editorials, case reports, conference reports, conceptual models, health programme descriptions

### Interviews

#### Sample selection

Our sample of researchers was drawn from the study database. Publications from the database were grouped by type of research (descriptive, intervention, reviews, other) and then by research project or topic area. Study authors selected the interview participants based on our sampling goals; that is, to include at least one author from each relevant body of research, plus representation from a variety of NSW research institutions. The policy decision-makers invited to participate in the study were employed by the NSW Ministry of Health or NSW Office of Preventive Health^1^ between 2000 and 2015. They held senior policy positions with responsibilities for childhood obesity policy development and implementation (director, branch manager or senior programme managers) and were selected based on their role and dates of employment to achieve our sampling goal of covering policy developments and key initiatives over the study time-period without gaps. Respondents were identified based on the research team’s knowledge and advice received from the NSW Ministry of Health. Thirteen researchers and fifteen policy respondents (decision makers) participated in the study, with a response rate of 81% (*n* = 13/16) and 88% (*n* = 15/17) respectively. Two of the research respondents and one policy respondent were co-authors of this paper, however they were not involved in the qualitative analysis.

#### Interview administration and questions

Interviews were conducted by telephone (by RN) between July and December 2016. Participants were provided with an interview guide (Additional file 1), including a list of identified research outputs categorized by research type, for research respondents. The interview schedules were piloted with the first respondents for each interview category. Piloting respondents completed the interview and then were asked to comment on the interview process and questions. No changes to the interview schedules were identified, so respondents participating in the pilot interviews were included in the study. Researchers were asked about their orientation towards achieving policy and practice goals, as well as reasons for conducting their listed research and the circumstances of its production. Decision makers were asked about their role in obesity prevention policy, the type of research investment and knowledge generation strategies that were used, and information available and used in decision making (Additional file 2). Researcher interviews took between 31 to 132 min (mean = 63 min) to complete, while decision-maker interviews ranged from 26 to 77 min (mean = 50 min).

#### Qualitative analysis

**Fig. 1:**
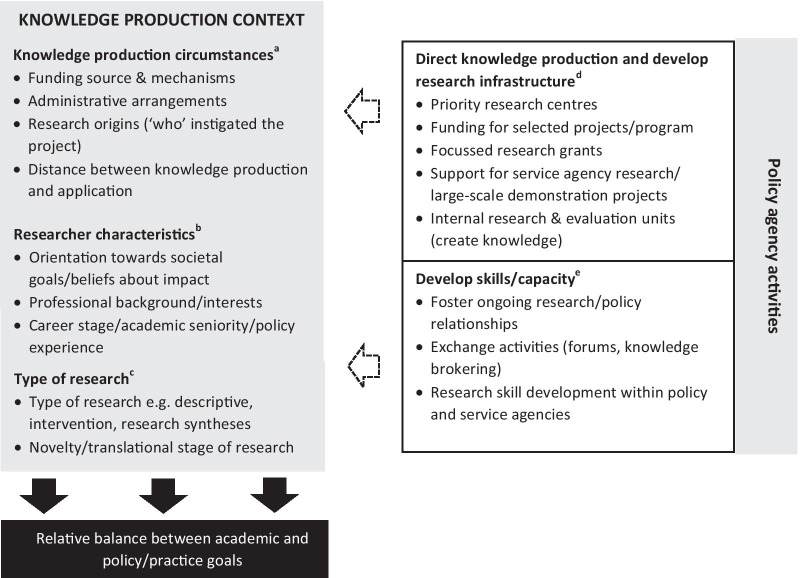
Factors influencing childhood obesity prevention research choices in NSW

All interviews were recorded and transcribed. The transcripts were checked for errors and uploaded into NVivo 11 Pro and coding was completed by RN. Inductive content analysis and a thematic approach was used to analyse the transcripts [42, 43]. Content was initially coded to an ‘intentions’ domain, which included references to why the research was conducted. The reasons given were coded as ‘academic reasons’ (associated with scientific enquiry, generating new knowledge, and academic system requirements) and ‘policy and practice reasons’ (practical application of the research considered from the outset), as these were the dominant categories identified when considering references within the intentions domain. In addition, content within each category was examined and compared by type of research. A final inductive cycle of coding of the interview transcripts was completed to consider emergent themes related to the context of knowledge production and how this influenced research choices. These themes are reported in the results and illustrated in Fig. 1.

## Results

### Research output database

In terms of peer reviewed publications, descriptive research publications were the most prevalent (42%; 228/543), followed by intervention research (26%; 140/543) and research syntheses (10%; 57/543;).^2^ The relative proportion of intervention research publications increased over the study time-period, possibly reflecting the demand for solutions to address childhood obesity. However, very few (1.3%; 7/543) publications reported on replication or dissemination studies. Most projects included multiple publications, so the number of outputs exceeds the number of interventions tested. Many intervention studies also included a literature review/s and descriptive analysis of study datasets so there were links between categories of research.

### Interviews

The 13 participating researchers [R1-13] contributed to 59% of the peer reviewed publications (*n* = 322/543). Participants worked at university-affiliated research institutes/centres [4], universities [3], local health districts [2] plus four others held health service positions with some university affiliation. Their professional backgrounds included health promotion practitioners and service managers, dietitians, teachers, clinicians and academics. Six participants described themselves as intervention researchers, and the remainder were broad-based, and all were experienced researchers in childhood obesity. As for policy respondents, six were employed at director level [P1-6] and nine as branch managers/senior programme manager [PM7-15]. There were overlaps in employment periods between the respondents, and as a group overall, their employment periods covered our study timeframe without gaps.

### Thematic analysis

#### Reported rationales for obesity prevention research include academic, policy and practice goals

Research respondents valued having an impact beyond knowledge creation and working purely in academic settings…*‘I guess it’s an intrinsic driver of why I’ve taken on the profession I have and why I do what I do’* [R9]*.* However, their research was conducted for mix of reasons, traditional academic inquiry as well as intentions to influence policy and practice, or a mix of both purposes.

Reported academic goals are shown in Table 1. Broader goals included intentions to expand the knowledge base or research field; while individually focussed goals included developing expertise, attaining qualifications, satisfying curiosity, developing personal areas of interest and meeting academic performance requirements. Academic goals were not often reported by policy respondents. Where reported, they reflected the value agencies placed on contributing to the knowledge base and staff attaining research skills and qualifications….*‘we are given opportunities if that's an area [research qualifications] we want to further in our role’* [PM13]*.*

**Table 1:** Examples of academic reasons for conducting research

Satisfy personal interest/curiosity e.g. ‘if you have a data set you can explore some interesting things’ [R11]
Build personal knowledge, develop expertise, leading to recognition and career advancement e.g. ‘it helps you understand where you might target things, and probably builds into a body of work that actually really helps’ [R7]
Meet publication expectations/targets e.g. ‘to some degree we are influenced by the publish or perish mentality’ [R5]
Support research students/assist in attaining postgraduate qualifications e.g. ‘it came out of PHD work….where you raise questions about things’ [R7]
Advance the field/contribute to new knowledge/innovate e.g. ‘with the PhD student…one part of it is thinking of unique novel ways and how they can advance the field’ [R11]
Add to the existing evidence base/consolidate knowledge e.g. ‘if we’re starting work in an area….we do like to start with a systematic review so you know what's happening in the field’ [R10]
Collaborate with other researchers/expand the research field e.g. ‘they were largely conducting research on xx. …..and came looking for my obesity expertise’ [R7]
Inform further research and funding applications/secure additional funding e.g. ‘we used this pilot to inform a funding application for a bigger study’ [R12]
Build on from previous research/improvement and progression within individual research portfolios e.g. ‘it was more about trying to understand the patterns and behaviour and the predictors of the behaviours that would help inform future versions of the interventions’ [R6]
Bring issues to the attention of other researchers e.g. ‘We’re try to inform the field…that descriptive research or pilot study may inform other people’s studies’ [R11]

Reported policy and practice goals are shown in Table 2. These were influenced by the relationships between the local research and policy systems. Where policy and practice goals were reported, researchers were trying to pre-empt policy needs, aiming to address local policy priorities or design interventions to ‘fit’ within local services; in some cases, after seeking advice from decision-makers and engaging them in the research process. They were aiming for their research to be *‘picked up on a larger scale and become mandated’* [R3]. There were also examples of research conducted ‘*to raise awareness of the issue amongst policy makers and then used as a call to action’* [R4]*.* The nature of goals reported varied by the type of research being conducted, reflecting the varying policy utility of different types of research (Table 2). Conversely, policy respondents reported looking to the local research system to meet their evidence generation needs. Investments in local research often focused on addressing evidence gaps or synthesizing existing evidence (local and international) to suit decision-making processes…*‘it was going to generate new knowledge and inform policy in the future’* [P2]. In addition, research was conducted to evaluate and improve existing services and for use as an advocacy tool in discussions with internal and external stakeholders.

**Table 2:** Examples of policy/practice focussed reasons for conducting/investing in research

Researcher perspective	Policy perspective
Descriptive research	Commissioned surveys/analysis of existing data sets
• Raise awareness of a problem/get issue on policy agenda e.g. association between soft drink consumption and obesity amongst NSW school children • Emphasize importance/priority of a problem to policy makers e.g. consequences of obesity; metabolic markers in obese teens • Counteract the influence of industry stakeholders on a policy process e.g. food industry; prevalence of junk food advertising during children’s viewing times • Build a case/advocate for specific a policy solution e.g. tougher advertising restrictions; greater emphasis on fundamental movement skills programmes • Identify and address gaps in understanding/knowledge that may aid in policy decision making e.g. stakeholder perceptions about obesity	• Define the problem in NSW; contribute to rationale for policy intervention e.g. local prevalence data • Convince stakeholders that there is a need for policy action; internal (within government) and external advocacy e.g. school-based data; active transport data • Monitor progress and reach of a strategy/programme • Highlight achievements; justify/seek ongoing investment • Plan policy initiatives and predict outcomes e.g. model intervention/ policy outcomes • Understand key settings and feasibility/acceptability of potential policy actions
Intervention research	Commissioned research/ programme evaluations
• Develop tools for practice e.g. programme materials • Develop a solution for known policy priority areas/settings e.g. childcare interventions • Design an intervention to fit with existing services with a view to wider scale-up e.g. obesity prevention intervention within home visiting services • Address a perceived gap in service delivery with a view to influence policy activity/inactivity in this area e.g. interventions for adolescents in schools • Build on from previous research to improve its relevance, fit, and sustainability for wider implementation at scale e.g. different iterations of the same intervention in schools • Provide policy relevant information in relation to an intervention e.g. economic analysis as part of a broader programme of research • Improve local services e.g. school canteen interventions within local health districts	• Generate evidence where no/limited evidence exists; trial new interventions in high priority policy areas e.g. childcare interventions • Address gaps in existing research knowledge; real world effectiveness, cost, feasibility/acceptability in relation to specific interventions • Improve the efficiency/quality of existing services e.g. test less resource intensive modes of service delivery; electronic reminders/incentives • Test interventions that have been trialled in other settings or locations to determine suitability/effectiveness within the local context e.g. group-based obesity treatment programmes • Determine the outcomes of existing programmes/services; inform future programme delivery e.g. childcare interventions • Test specific approaches/ideas e.g. population wide approaches
Research syntheses	Commissioned literature reviews
• Provide advice for practitioners; best practice approaches • Understand an issue before engaging with policy makers e.g. links between soft drinks, weight status and health • Highlight gaps in government policy response; advocate for further/alternate/new strategies • Advocate for further research in an area; influence research funding strategies e.g. demonstrate a gap in knowledge exists	• Provide rationale for policy action and policy choices; for use as an internal and external advocacy tool; to support specific policy processes (action plan development, consultation process); to feed into decisions about continued investment/changes • Guide/inform practice e.g. development of practice-based guidelines • Understand new areas for potential policy intervention e.g. technology-based interventions • Determine ‘best buys’, most promising areas for intervention; understand strength of evidence in relation to different options • Inform programme/service development; what to do and how to do it in relation to a specific intervention • Identify areas requiring further research in relation to policy information gaps

#### Knowledge production circumstances influence research choices

Contextual and other influences on childhood obesity research are shown in Fig. 1. The circumstances of knowledge production (Fig. 1; a), the characteristics of researchers involved in a project(Fig. 1; b) and the type of research conducted (Fig. 1; c) all contributed to the relative balance between academic and policy and practice focussed research goals. Interviews also showed that policy agencies instigated a range of activities to support research on obesity prevention (Fig. 1; d–e), contributing to policy relevant knowledge production in NSW.

#### Knowledge production circumstances

**Fig. 2:**
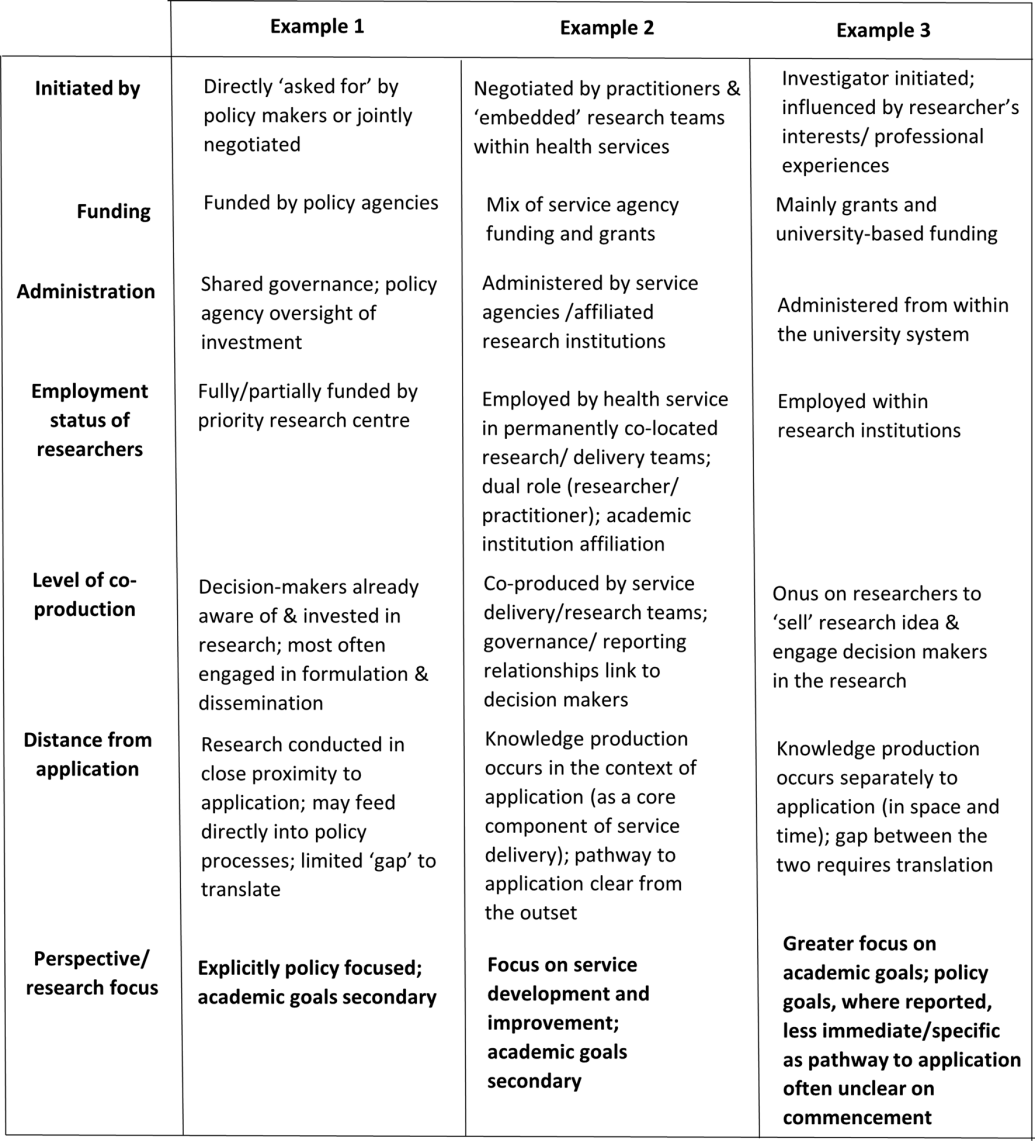
Perspective from which childhood obesity prevention knowledge is produced by knowledge production circumstance

The three main examples of knowledge production circumstances and mechanisms derived from descriptions provided by study respondents are illustrated in Fig. 2. These examples differ in terms of their funding mechanisms, administrative arrangements, ‘who’ instigated and participated in the research, and the relative distance between knowledge production and its application (Fig. 1; a). The different circumstances of knowledge production described by respondents provided different incentives and created different requirements, that interacted to influence the reported goals of the research. For instance, in Example 3, where primary investigators were employed in public research institutions and the research was investigator initiated, academic goals featured more prominently. In this example, the interests of individual researchers were the primary drivers of research choices. Also, in Example 3, research system performance measures incentivized academic goals such as peer reviewed publication and conducting research to inform funding applications. Policy focussed goals were reported but these were less immediate and specific than for the other examples included within Fig. 2. In the other examples the primary drivers were the research interests of health service employers (Example 2), or the needs of policy agencies as the primary research funders (Example 1), and in these examples service improvement and policy focussed goals predominated (Fig. 2).

The policy agencies included in our study strongly influenced policy-relevant knowledge production in NSW by directing research activity and contributing to research infrastructure development (Fig. 1; d). They supported priority research centres, large research projects and focussed grant schemes that had an explicit policy focus. Priority research centres completed a negotiated work programme, addressing medium to long term evidence needs (see Example 1; Fig. 2)….. ‘*it was originally conceived… so that if nothing else happened we had evidence generation and we had some surveillance happening that would keep the issue on the agenda*’ [P2]. In addition, specific project support was provided for large projects or to address acute policy needs. Finally, policy agency administered research grants supported research rarely funded by other grant schemes, with selection criteria weighted towards potential scalability and co-production….‘w*e're making it explicit to them that, really, translation is front and centre about this whole process*’ [P6].

Another mechanism that supported policy and practice focussed knowledge production in NSW was the embedding of researchers/research teams within service agencies (Fig. 1; d). In some cases the research was conducted by ‘*an integrated research and practice unit’* [R9] permanently embedded within the service agency and responsible for service development as well as research, as described in Example 2 (Fig. 2) … ‘*we embarked on trials basically to improve how we deliver services’* [R9]; ‘*it’s hard to say which team started the research, whether it’s the implementation team or evaluation team*’ [R3] Variations of these circumstances included situations where research was investigator-initiated but research staff were temporarily employed within service agencies, conducting research as a separate process, ‘parallel to’, rather than integrated into core service delivery. These circumstances differed from Example 2 in that the distance between knowledge production and its application, the ‘gap’ requiring translation was increased, and this influenced the nature of policy goals reported.

#### Researcher characteristics that influence research choices

While individual researchers within our sample had similar orientations towards societal goals, they differed in terms of their beliefs about impact, professional backgrounds and policy experience, and these differences influenced their research choices (Fig. 1; b). In terms of beliefs about impact, researchers who defined policy impact as the direct adoption of their own research pursued goals along those lines. Whereas some researchers felt their research should contribute to the broader evidence base, rather than directly influencing policy. These researchers focused on contributing to policy debate and improving policy approaches in general, putting their research into context with other evidence when engaging with decision makers…*‘we used it as a vehicle……to bring to their attention the recent evidence from that study or particular review’* [R13]*.*

Differences in professional background (e.g. health promotion, education, clinical) and policy experience manifested in choices of research topic, the type of research conducted and the policy sector and level (e.g. local, national, international) within which researchers engaged. Policy experience was linked to experience in the research area, with respondents reporting their policy relationships and engagement skills had developed over time ….*‘you get known… and over a decade, it becomes a way of working. It's not an instant thing’* [R13]*.* In addition, career progression and recognition of expertise led to opportunities to conduct policy relevant research (e.g. commissioned research)….*‘other research has been serendipitous, I guess just with our reputation in the area….being approached by the World Health Organisation*’ [R4].

Policy agency research engagement and capacity building activities also influenced researchers within this research-policy system (Fig. 1; e). The development of long-term relationships between researchers and decision makers, fostered an understanding of policy processes and needs amongst this group of researchers. In addition, activities to develop internal research capacity meant that policy makers or practitioners attained research qualifications, creating a diversity of skill sets within the research-policy system.

#### Type of research

There were differences in the relative focus of policy and academic goals depending on the type of research being conducted (Fig. 1; c) and this was linked to ‘who’ initiated the research (Fig. 2). Overall, policy goals were less commonly reported for descriptive research or research syntheses than for intervention research. Much of the descriptive research and research synthesis conducted with policy goals in mind was driven by the needs of policy/service agencies.

Policy goals were common drivers of intervention research, but there were differences in how specific and immediate these were, depending on who had initiated the research. Policy and service agencies predominantly invested in the later translational stages of intervention research (e.g. implementation and dissemination research) to inform policy and programme decisions…… ‘*generally what we do is we design interventions or we take interventions that have already been designed or best practice guidelines, and we apply them locally’* [R9]. Researchers conducting investigator initiated research often began with new research topic areas or interventions seeking to progress these and then make their research more suited to the local policy context…* ‘I keep my eye on all the literature around what is and what's isn’t been done around the world, and I always try to be innovative’* [R6]. Here, the pathway for application of the research was not always clear until the direction of the research findings were known, so their goals were less immediate and specific.

However, researchers reported a strong sense of the relative connections between research across portfolios. Descriptive research and research syntheses were often linked to intervention research. In some cases, descriptive studies generated information to understand ‘*what was going on*’ [R6] or to help understand behaviours and this fed into future intervention design. Similarly, research syntheses were conducted to decide which direction to take in relation to future research…*‘what do we know about this area, do we really need to keep working this area or are there other issues that require more attention?’* [R1]*.* In addition, different types of research were seen to contribute to development of personal knowledge and expertise, influencing individual credibility and recognition, which in turn facilitated policy influence……*‘really my original childhood obesity work has led me to be able to do those studies and make much stronger calls…. at both state and national levels’* [R10].

## Discussion

This study examined why childhood obesity prevention research was conducted, and the factors influencing research choices from the perspective of involved researchers and health policy agencies. Our findings suggest that this group of researchers were intrinsically motivated to make a difference beyond simply creating new knowledge. However, some research was conducted entirely for traditional academic reasons. Reported goals varied between research projects and across research portfolios, influenced by factors both external to individual researchers, and those associated with the characteristics and experience of the individuals involved. Different knowledge production contexts and circumstances influenced the perspective from which knowledge was produced, and subsequently the reasons research was conducted. These findings are consistent with the framework reported by D’Este et al. [19], describing individual antecedents, organisational conditions, and processes encouraging productive interactions as factors influencing societal versus academic research goals [19]. The current study provides empirically derived insights regarding the interaction of these factors in practice. [19].

Unlike previous studies, this research includes the perspective of researchers employed outside of public research institutions, as well as those of decision makers from health policy agencies who funded and contributed to research [19, 30]. The range of perspectives provided reveal a shift, both over time and in knowledge production context, from a focus on curiosity-driven scientific inquiry (mode 1 approaches) to approaches where greater emphasis is placed on the policy and societal value of research (mode 2 approaches) [29]. Several factors contributed to this shift in this study. Firstly, knowledge was produced within a wide range of organisations and by actors with a diversity of skill sets and professional experiences [29]. Secondly, different levels of co-production of research were evident, sometimes with limited distinction between producers and users, indicating a blurring of traditional lines between research and policy communities [11, 33]. Finally, there was a level of ongoing linkage and engagement across the policy and research sectors, contributing to policy focussed research [11]. This meant there was not only a one-way transfer of new knowledge from researchers, through dissemination, to end users, but bi-directional knowledge flows and contributions to research and policy processes [24].

There were still some tensions between ‘scientific’ and ‘societal’ goals of childhood obesity prevention research conducted within NSW. In particular, tensions arose between the pragmatic answers required by policy makers and efficacy and evidence generation important to researchers [7, 8]. Researchers also reported that the connections between different types of research and their role in understanding a topic were important. They considered that preliminary research, with an academic focus, wasn’t ‘wasteful’ but contributed to policy influence over time. This finding reinforces the observation that it is usually a programme of research that has policy influence rather than any individual project [44]. It also supports the position that research driven by policy and societal goals should complement, rather than replace curiosity-driven research [29]. However, moving existing interventions forward on the intervention research continuum rather, than starting at the beginning, in the name of innovation, will accelerate the speed with which translation occurs [45]. Researchers working in both modes simultaneously and the pull back to mode 1 approaches in defence of research quality and hierarchical evidence structures have been reported in other jurisdictions [30].

In terms of push and pull mechanisms, our findings highlight how funding mechanisms and opportunities influenced research choices. At the time of this study grants within the Australian research system focused on short term investigator driven projects rather than complex, long-term studies. Newer funding mechanisms such as Centres of Excellence and Partnerships Grants were only starting to have an influence towards the end of our study period. Producer-push funding strategies, led by research funding bodies, therefore played a limited role in facilitating the production of policy relevant research in this instance. However, the user-pull strategies employed by policy agencies were influential (Fig. 1). In particular, funding priority research centres and grant schemes supporting translational research were important and demonstrate the value of this type of funding within research systems.

The close relationship between the policy context and research goals in this case raises questions about what is more desirable: for policy priorities to direct research or for research to direct policy priorities? Childhood obesity prevention was the ‘flavour of the decade’ in NSW during our study timeframe. Policy agenda setting exercises (e.g. NSW Childhood Obesity Summit) resulting in government action plans and their ongoing revisions, influenced the activities of researchers in NSW. However, once policy trajectories were established, research tended to contribute to solving the policy issue rather than stimulating policy agendas. This highlights how policy choices may be sustained in some cases, even when there is evidence for alternate approaches. It also meant that alternative public health policy priorities and research topics received less attention. For example, there was little policy or research attention on weight gain during early adulthood, the decade where maximal weight gain occurs [46]. In addition, conducting research with a policy focus helps make policy evidence informed, but this doesn’t mean the research or policy questions are answered, or that the policy is strong, comprehensive or uncontested.

### Study limitations

This case study may not be representative of all research or researchers. These findings relate to childhood obesity research, and in the context of the state of New South Wales, which has had a strong tradition of population health and prevention research. Hence, these findings are not necessarily generalisable to other contexts. Our sampling strategy resulted in researchers who had completed a variety of research projects with different affiliations being invited for interview. As a result of this strategy, our research respondents were experienced researchers. They were also a highly productive group of researchers, contributing to 59% of the identified peer reviewed research outputs in NSW between 2000 and 2015. These characteristics and the applied nature of the research topic may have influenced their perspectives. In addition, the study was conducted at a time when research impact, although not a performance metric, was being widely debated, possibly contributing to reported motivations.

In terms of our choice of study location and policy area, the relationship between the research and policy sectors in this case may be atypical, as childhood obesity was a priority policy and research area during the study period. The growth and stability of the policy and research topic may have influenced the reciprocal understanding between players that was observed. Although we note some similarities in our findings with studies conducted in other jurisdictions and literature reviews on the topic [19, 27, 30, 31, 33].

Another limitation of this study is that the literature search strategy used to identify research may not have identified outputs if the research was conducted in NSW, but none of the authors had an affiliation in NSW. However, we consider it unlikely that research could have been conducted in NSW without input from NSW researchers. This limitation may be an issue if the method employed in the current study was to be replicated in low to middle income counties, where ‘in-country’ researchers and research institutions may be absent.

## Conclusions

In this case study we found that Australian childhood obesity researchers undertook research with a mix of motivations related to both traditional academic reasons and a desire to influence or contribute to policy-related priorities. They were strongly influenced by their work environment and factors associated with the knowledge production context. Our findings indicate that a shift towards mode 2 research approaches is happening in practice in this case. Researchers are taking on societal goals and are aware of and engaging in translational actions where practical. These actions have been facilitated by measures taken by policy agencies to direct policy relevant knowledge production. But this shift does not answer all policy questions, introduces opportunity costs (directing research away from other research questions), and does not ensure research findings address policy issues in a timely manner. It is imperfect and complex to get these systems to enhance each other, however in this instance, this goal is being actively and constructively pursued by the childhood obesity prevention research and policy communities in NSW.

It is acknowledged that this case study may have distinctive characteristics, such as the long-term local research-policy linkages. In many ways the constellation of contextual factors, related to the topic being a policy priority, policy-driven funding of research and the embedded nature or strong connections between many of the researchers and service/policy agencies, make it a rather exemplary example. This study suggests that research systems should focus on structural solutions, intervening before knowledge is produced, to foster long term collaborations between policy and research groups rather than focussing on the dissemination efforts of individual researchers.

## Supplementary Information

**Additional file 1.** Researcher interview guide.

**Additional file 2.** Decision maker interview guides.

## Data Availability

The datasets used and/or analysed during the current study are available from the corresponding author on reasonable request.

## Footnotes

1 The NSW Ministry of Health, Population Health Division (also known under other names during the study period) and NSW Office of Preventive Health, were the policy agencies primarily responsible for child obesity policy development and implementation in NSW between 2000 and 2015.

2 Measurement studies (4%; 22/543) and other publications (e.g. editorials, commentaries, policy/programme descriptions and analyses) (18%; 96/543).

## References

[1] Oliver K, Innvar S, Lorenc T, Woodman J, Thomas J (2014). A systematic review of barriers to and facilitators of the use of evidence by policymakers. BMC Health Serv Res.

[2] Innvaer S, Vist G, Trommald M, Oxman A (2002). Health policy-makers' perceptions of their use of evidence: a systematic review. J Health Serv Res Policy.

[3] Hanney SR, Gonzalez-Block MA, Buxton MJ, Kogan M (2003). The utilisation of health research in policy-making: Concepts, examples and method of assessment. Health Res Policy Syst..

[4] Caplan N (1979). The two-communities theory of knowledge utilisation. Am Behav Sci..

[5] Weiss CH (1979). The many meanings of research utilization. Public Adm Rev.

[6] Campbell DM, Redman S, Jorm L, Cooke M, Zwi AB, Rychetnik L (2009). Increasing the use of evidence in health policy: practice and views of policy makers and researchers. Aust New Zealand health policy.

[7] Milat AJ, Bauman AE, Redman S, Curac N (2011). Public health research outputs from efficacy to dissemination: a bibliometric analysis. BMC Public Health.

[8] Sanson-Fisher RW, Campbell EM, Htun AT, Bailey LJ, Millar CJ (2008). We are what we do: research outputs of public health. Am J Prev Med.

[9] Wolfenden L, Wiggers J, Tursan d'Espaignet E, Bell AC (2010). How useful are systematic reviews of child obesity interventions?. Obes Rev.

[10] Dobrow MJ, Miller FA, Frank C, Brown AD (2017). Understanding relevance of health research: considerations in the context of research impact assessment. Health Res Policy Syst.

[11] Mitchell P, Pirkis J, Hall J, Haas M (2009). Partnerships for knowledge exchange in health services research, policy and practice. J Health Serv Res Policy.

[12] Ladd JM, Lappe MD, McCormick JB, Boyce AM, Cho MK (2009). The "how" and "whys" of research: life scientists' views of accountability. J Med Ethics.

[13] Haynes AS, Derrick GE, Chapman S, Redman S, Hall WD, Gillespie J (2011). From "our world" to the "real world": Exploring the views and behaviour of policy-influential Australian public health researchers. Soc Sci Med.

[14] Moher D, Naudet F, Cristea IA, Miedema F, Ioannidis JPA, Goodman SN (2018). Assessing scientists for hiring, promotion, and tenure. PLoS Biol.

[15] Rice DB, Raffoul H, Ioannidis JPA, Moher D (2020). Academic criteria for promotion and tenure in biomedical sciences faculties: cross sectional analysis of international sample of universities. BMJ.

[16] van der Weijden I, Verbree M, van den Besselaar P (2012). From bench to bedside: the societal orientation of research leaders: The case of biomedical and health research in the Netherlands. Sci Public Policy.

[17] Wilson PM, Petticrew M, Calnan MW, Nazareth I (2010). Does dissemination extend beyond publication: a survey of a cross section of public funded research in the UK. Implement Sci.

[18] Brownson RC, Jacobs JA, Tabak RG, Hoehner CM, Stamatakis KA (2013). Designing for dissemination among public health researchers: findings from a national survey in the United States. Am J Public Health.

[19] D’Este P, Ramos-Vielba I, Woolley R, Amara N (2018). How do researchers generate scientific and societal impacts? Toward an analytical and operational framework. Sci Public Policy.

[20] Tabak RG, Stamatakis KA, Jacobs JA, Brownson RC (2014). What predicts dissemination efforts among public health researchers in the United States?. Public Health Rep.

[21] Campbell D, Moore G (2018). Increasing the use of research in population health policies and programs: a rapid review. Public Health Res Pract.

[22] La Brooy C, Kelaher M (2017). The research-policy-deliberation nexus: a case study approach. Health Res Policy Syst.

[23] Greenhalgh T, Jackson C, Shaw S, Janamian T (2016). Achieving research impact through co-creation in community-based health services: literature review and case study. Milbank Q.

[24] Holmes BJ, Best A, Davies H, Hunter D, Kelly MP, Marshall M (2017). Mobilising knowledge in complex health systems: a call to action. Evid Policy.

[25] Wolfenden L, Yoong SL, Williams CM, Grimshaw J, Durrheim DN, Gillham K (2017). Embedding researchers in health service organizations improves research translation and health service performance: the Australian Hunter New England Population Health example. J Clin Epidemiol.

[26] Jansen MWJ, van Oers HAM, Middelweerd MDR, van de Goor IAM, Ruwaard D (2015). Conditions for sustainability of Academic Collaborative Centres for Public Health in the Netherlands: a mixed methods design. Health Res Policy Syst.

[27] Lavis JN, Lomas J, Hamid M, Sewankambo NK (2006). Assessing country-level efforts to link research to action. Bull World Health Organ.

[28] McLean RKD, Graham ID, Tetroe JM, Volmink JA (2018). Translating research into action: an international study of the role of research funders. Health Res Policy Syst.

[29] Hessels LK, van Lente H (2008). Re-thinking new knowledge production: a literature review and a research agenda. Res Policy.

[30] Ferlie E, Wood M (2003). Novel mode of knowledge production? Producers and consumers in health services research. J Health Serv Res Policy.

[31] Newton MS, Estabrooks CA, Norton P, Birdsell JM, Adewale AJ, Thornley R (2007). Health researchers in Alberta: an exploratory comparison of defining characteristics and knowledge translation activities. Implement Sci.

[32] Pham J, Pelletier D (2015). Action-oriented population nutrition research: high demand but limited supply. Glob Health Sci Pract.

[33] Jansen MW, De Leeuw E, Hoeijmakers M, De Vries NK (2012). Working at the nexus between public health policy, practice and research Dynamics of knowledge sharing in the Netherlands. Health Res Policy Syst..

[34] Nathan SA, Develin E, Grove N, Zwi AB (2005). An Australian childhood obesity summit: the role of data and evidence in 'public' policy making. Aust New Zealand Health Policy..

[35] King L, Turnour C, Wise M (2007). Analysing NSW state policy for child obesity prevention: strategic policy versus practical action. Aust New Zealand Health Policy.

[36] Develin L (2004). Measures taken in New South Wales to address childhood obesity following the NSW Childhood Obesity Summit. NSW Public Health Bull.

[37] NSW Health. Premiers Priority Childhood Obesity Delivery Plan. 2016. https://www.health.nsw.gov.au/heal/Publications/Premiers-priority-childhood-obesity-delivery-plan.pdf. Accessed Jan 2021.

[38] NSW Deaprtment of Health (2010). Promoting the generation and effective use of population health research in NSW. A Strategy for NSW Health 2011–2015.

[39] NSW Department of Health. Prevention of Obesity in Children and Young People: NSW Government Action Plan 2003–2007. http://web.archive.org/web/20080725091243/http://www.health.nsw.gov.au/obesity/adult/gap/ObesityActionPlan.pdf. Accessed Jan 2021.

[40] Baur LA, Wake M, Espinel PT (2010). Letters to the editor. J Paediatr Child Health.

[41] Lubans DR, Jones R, Okely AD, Salmon J, Baur LA (2013). Review of Australian childhood obesity research funding 2010–2013. Health Promot J Austr..

[42] Thomas DR (2006). A general inductive approach for analyzing qualitative evaluation data. Am J Eval.

[43] Nowell LS, Norris JM, White DE, Moules NJ (2017). Thematic analysis: striving to meet the trustworthiness criteria. Int J Qual Methods.

[44] Smith KE, Katikireddi SV (2013). A glossary of theories for understanding policymaking. J Epidemiol Community Health.

[45] Shelton RC, Lee M, Brotzman LE, Wolfenden L, Nathan N, Wainberg ML (2020). What is dissemination and implementation science? An introduction and opportunities to advance behavioral medicine and public health globally. Int J Behav Med.

[46] Munt AE, Partridge SR, Allman-Farinelli M (2017). The barriers and enablers of healthy eating among young adults: a missing piece of the obesity puzzle: a scoping review. Obes Rev.

