# Supplementary material for: The how and why of producing policy relevant research: perspectives of Australian childhood obesity prevention researchers and policy makers
Source: Health Res Policy Syst. 2021 Mar 10;19:33. doi: 10.1186/s12961-021-00687-0 (PMC7945318; doi:10.1186/s12961-021-00687-0)
Supplement: Supplementary file 1 — Additional file 1. Researcher interview guide. [file 12961_2021_687_MOESM1_ESM.docx]

**Supplementary File 1: Researcher interview guide**

**Measuring the Impact of Research on Childhood Obesity Policy in NSW between 2000 and 2015**

**INTERVIEW GUIDE – NSW Childhood Obesity Researchers**

*Hello my name is [insert research assistant name] and I am calling on behalf of the Prevention Research Collaboration, University of Sydney. Could I please speak with [insert interviewee name] regarding a telephone interview he/she has agreed to.*

If unavailable, organise to call back at an appropriate time.

If participant answers phone –

*Hello [insert interviewee name], my name is [insert research assistant name] and I am calling on behalf of the Prevention Research Collaboration, University of Sydney. Thank you for agreeing to participate in an interview examining the policy and practice impact of your childhood obesity related research.* ***Is now still a good time to do the interview?***

IF NO, organise an alternative time.

***Can you confirm whether you received the information sheet about the study and an outline of the interview questions?***

IF NO, organise to email through a copy and allow time for the participant to read the information sheet and ask any questions prior to proceeding with the interview.

**IF YES, *do you have any questions you would like to ask before proceeding?***

Interviews will be approximately 40 minutes. ***Would it be OK to tape record this interview for the purposes of analysis?***

Today I was hoping to hear about your views on the policy and program impacts of your childhood obesity research and on research impact more generally. I’ll also ask you some questions about the childhood obesity research infrastructure and networks in NSW and elsewhere. We wanted to interview you because when we completed a search for childhood obesity research conducted in NSW between years 2000 and 2015 we found that you had quite a substantial body of work in this area. I have made an attempt to summarise your work to guide our discussions today. I sent you this so it might help if you had it in front of you while we talk.

***Do you have this information in front of you?***

*If Yes, great, I will refer to this during our discussion.*

***If no, ok, do you mind if I email this to you now as it will aid our discussion?***

This might not be a complete summary of your work but I’ll ask you some questions about this later in the interview.

1. Firstly, I wanted to ask you about how important it is, to you, for your research to have policy and program impacts relative to other types of impacts eg on other research and publications?
   - What is your understanding of policy impacts?
   - Clarify definition we are using if necessary
2. What about where you expect to have policy and program impacts? Would you expect these to occur in NSW or elsewhere?
3. What do you do, if anything, to try to increase the impacts of your work on policy and programs?
4. Now going back to your body of work, the publications we identified seemed to be related to a number of key studies, X, Y Z and some other work …………………(provide a 1-2 sentence summary of the work) Is this correct?
5. We are interested in how you decided what work to complete or how these studies came about. Can you tell me a bit about this, starting with XXXX; what about xxxxxx?
   - prompt for grants/funding sources,
   - co-production/links with NSW health,
   - relationship to other research (local/international),
   - links between projects
   - types of publications (intervention/descriptive/research synthesis)
   - policy priorities
   - interests of other stakeholders

(Ensure all key projects are covered)

1. Was any of your work particularly impactful on policy and programs in NSW?
   - Which studies?
   - How would you describe the impact it had? (help understand issues; influenced program/policy content directly; persuade/symbolic).
   - Why/how did the impact occur?
   - What role do you think you played directly in these impacts?
   - How do you know these impacts occurred?
2. What about policy and program impacts outside of NSW?
   - Which studies?
   - Why/how?
3. Were there any studies you felt should have policy or program impacts in NSW but didn’t?
   - Why do you think this was?
4. What about the other research in the table that we haven’t already talked about?
   - Where does this research fit in terms of research impact?
   - How would you describe the value of this research? (prompt with specific examples for discussion)
5. Is there any of your work we haven’t captured that you think is policy relevant?

**Research infrastructure questions**

I’m also interested in the childhood obesity research ‘system’ more broadly in NSW and elsewhere.

1. Who are the key groups or individuals involved in childhood obesity research in NSW?
   - Who is involved?
   - What role do they play?
   - How are these groups organised?
   - prompt if necessary – what about the NSW Ministry of Health – what role does it play?
   - What sort of mechanisms, if any, are in place to encourage engagement between the various stakeholders involved?
   - To what extent are you or your research institution involved in these activities?
2. What about in Australia? Internationally?
   - Who is involved?
   - What role do they play?
   - How are these groups organised?
   - Are there any links between these groups?
   - What sort of mechanisms, if any, are in place to encourage engagement between the various stakeholders involved?
   - To what extent are you or your research institution involved in these activities?
3. Is it alright if I contact you again if anything comes up during the remaining interviews that I’d like to clarify with you?

*We have come to the end of the questions. Thank you for your time today. Your insights have been very useful***.**

**Definitions (if asked by respondents to define a term)**

**Policy and program impacts …**Policies are statements or actions taken by government in response to a particular problem or issue. Your research may have had an impact on policies and programs in a direct or instrumental way such as contributing directly to the design and implementation of a particular program or initiative or in more subtle ways such as influencing thinking on an issue or being used to support a pre-existing policy position or program that is already being implemented. Or you may know that your research has been cited in a particular policy document such as a report, review, action plan, implementation plan, protocol or guideline. We are interested in any and all of these types of impacts

**Research is...**Analyses of quantitative or qualitative data, or theory, found in peer reviewed papers, technical monographs or books, or in grey literature such as internal studies and evaluations, and reports on authoritative websites. Advice from researchers is considered to be research‐informed information, but not research per se. We use the terms research, research findings and research evidence interchangeably.

**Policies are...**A formal statement or action developed by a government agency or statutory body in response to an identified problem. This includes state‐wide or national legislation, policies, programs, directives, protocols, guidelines, and service models.

**A policy document is**...A review, report, discussion paper, draft or final policy, formal directive, program plan, strategic plan, ministerial brief, budget bid, service agreement, implementation plan, guideline or protocol with a focus on health service or program design, delivery, evaluation or resourcing.

**Intervention research is**.…research that examines the impact of an intervention (whether it does more good than harm in a target population). The research can be delivered under optimal conditions or in real world conditions. It may assess the practicality of implementing interventions with demonstrated efficacy in new populations or settings or may assess the wide scale use of an intervention across a target population or whole population.

**Descriptive research is**…data based research that describes the nature and scope of the problem and develops solutions using qualitative or quantitative methods. This type of research may explore the frequency, patterns, correlates or predictors of obesity or related variables such as knowledge, attitudes, healthcare practices, policies or legislation. It also includes etiological studies that investigate the casual relationship between exposure to a risk factor and subsequent illness, disease or health outcome.

**Research synthesis is**…a synthesis of existing research that may take the form of meta analyses, systematic reviews or literature reviews.

We have excluded clinical and biomedical research from our sample as our focus is on population health policy and research.
