# Supplementary material for: The how and why of producing policy relevant research: perspectives of Australian childhood obesity prevention researchers and policy makers
Source: Health Res Policy Syst. 2021 Mar 10;19:33. doi: 10.1186/s12961-021-00687-0 (PMC7945318; doi:10.1186/s12961-021-00687-0)
Supplement: Supplementary file 2 — Additional file 2. Decision maker interview guides. [file 12961_2021_687_MOESM2_ESM.docx]

**Supplementary File 2: Decision maker interview guides**

**Measuring the Impact of Research on Childhood Obesity Policy in NSW between 2000 and 2015**

**INTERVIEW GUIDE – Policy makers**

**INTERVIEW QUESTIONS**

1. Can you tell me the timeframe you have been/were involved in childhood obesity policy development and or implementation in NSW?
2. What was your role during this time? Did your role change at all?
   - *(NB: if multiple roles, remember to ask whether perspective change overtime when discussing the questions below).*
3. What was happening and what factors were at play?
   - *The political ideology and priorities? Ministers priorities?*
   - *Legislative and policy infrastructure?*
   - *Availability of resources and funding? Overall economic climate?*
   - *Extent of media involvement and interest?*
   - *Public opinion?*

*(probe for role played, extent and direction (positive/negative) of influence)*

1. What place did research have in this process? Where did research fit in with what you have described?
   - *Was there any requirement to consider research evidence? (not a great deal; loosely encouraged; expected to use research; mandatory use)*
   - *What was the prevailing attitude and organisational culture towards research use?*
   - *What about the capacity and skills of staff in relation to research use?*
   - *What influence did this have?*
   - *Did this change over time? What led to any changes? And what impact did any change have?*
2. Did you/your centre use any specific strategies to try to find out about research or engage with researchers?
   - *What was done?*
   - *Which groups or individuals specifically?*
   - *How successful were these strategies?*
   - *What influence did these strategies/relationships have?*
3. What can you remember about the type of research that was available at the time?
   - *Extent, quality and relevance of the available research?*
   - *Overseas, Australian research, local/NSW research?*
   - *Content: Causes of obesity? Prevalence? Solutions? Who to target? How to implement solutions?*
   - *New or old research?*
   - Were there any gaps in terms of information you needed that wasn’t available?*What did you do about these gaps? Commissioning research?*
   - *Influence this had on the policy process?*
   - *Changes over time?*
4. Where any specific studies or programs of research particularly influential?
   - *Which research? (Overseas, Australian research, local/NSW research? Causes of obesity? Prevalence? Solutions? Who to target? Implement solutions? Old/new?*
   - *Why was this research useful? How was this research used? (Help to understand or think about the issue? Make decisions about the program policy content and direction? Persuade others to a point of view or course of action? Support a predetermined position? )*
   - *Comment on the relative use and value of international compared to local research?*
   - What about specific researchers or research groups? Was any one particularly influential? *Which researchers/groups? What was the nature of their influence?*
   - *Extent of influence on the policy decision-making process in comparison to other factors described? (virtually none; very little influence; some influence; or substantial/extensive influence).*
5. Did anything we haven’t already talked about have an influence on the use of research in policy decisions during this time?
6. Is it alright if I contact you again if anything comes up during other interviews that I’d like to clarify with you?

**Measuring the Impact of Research on Childhood Obesity Policy in NSW between 2000 and 2015**

**INTERVIEW GUIDE – Program managers**

**INTERVIEW QUESTIONS**

I’m interested in learning about your role in the [insert name program/initiative] and whether and how research was used to inform the development of implementation of this program/initiative?

1. Can you tell me how you have been involved in this program/initiative and over what time frame?
   - Involvement in development, implementation or both?
2. Can you tell me a little bit about this initiative?
   - When did it commence?
   - How did it come about?
   - How is it funded?
   - Who is responsible for its implementation?
   - Who else is involved? (key stakeholders, experts)
3. (If not already mentioned) Did research inform the development or implementation of this initiative in any way?
   - What research specifically? (international/local; research synthesis/primary studies; descriptive/intervention research)
   - What role did it play? (increase awareness/conceptual, direct/instrumental, symbolic/persuasive)
   - Type of information the research provided (causes; prevalence; solutions; how to implement; who to target; costs)
   - How was it used? Specific examples.
   - Recent/old research?
   - Quality/relevance of the research
4. How did you find out about the research?
   - Specific strategies used to engage with research/researchers?
   - Specific groups or individuals?
5. Was there information/research you needed that wasn’t available? How did you deal with this?
   - Commission, conduct or advocate for more research?
6. How would you rate the importance of research in the development and implementation of this initiative? Compared to the other factors involved and other information sources?
7. How was the use of research to inform policy viewed within your organisation or division at the time?
8. What about your personal views about research use?
   - Do you think it is important?
   - How much emphasis should be placed on it?
   - Have your views changed over time?
9. Was research cited in any reports or policy documents associated with this initiative/program? What relationship does the cited research have to overall body of research you considered?
10. Do you have anything further to add about the use of research in in relation to this initiative/program?
